# Simultaneous Estimation of Andrographolide and Wedelolactone in Herbal Formulations

**DOI:** 10.4103/0250-474X.45421

**Published:** 2008

**Authors:** M. B. Patel, V. M. Kadakia, S. H. Mishra

**Affiliations:** Pharmacy Department, Kalabhavan, Faculty of Technology and Engineering, The M. S. University of Baroda, Vadodara-390 001, India

**Keywords:** Andrographolide, wedelolactone, *Andrographis paniculata*, *Eclipta alba*, hepatoprotective agents, HPTLC

## Abstract

Andrographolide and wedelolactone are active components of *Andrographis paniculata* and *Eclipta alba,* respectively. The extracts of these plants are used in many traditional hepatoprotective formulations. An attempt has been made to develop an accurate, precise and specific HPTLC method to quantify simultaneously both these chemical markers of diversified chemical structures in different dosage forms like tablet and syrup. Precoated silica 60F_254_ plates with toluene:acetone:formic acid (9:6:1) as mobile phase and detection wavelength of 254 nm were used. The method was validated in terms of linearity, accuracy, precision and specificity. The calibration curve was found to be linear between 200 to 400 ng/spot for andrographolide and 100 to 200 ng/spot for wedelolactone. The limit of detection and the limit of quantification for andrographolide were 26.16 and 79.28 ng/spot, respectively and for wedelolactone 5.06 and 15.32 ng/spot, respectively.

Herbal medicines are generally available as a mixture of more than one plant constituent. It is important to quantify the maximum possible number of markers in such herbal formulations through which the quality of the formulations may be assessed / assured. *Andrographis paniculata* (Acanthaceae) commonly known as *Kalmegh,* is widely used in Indian traditional system of medicine as one of the remedies to treat diseases related to liver. The drug is mainly recognized due to the presence of a diterpenoids, andrographolide and related compounds^1^. The plant has been successfully evaluated for various liver protective activities^2^–^6^. Andrographolide and related compounds have shown varying degrees of antipyretic, antimalarial, antiinflammatory and antihepatotoxic effect^7^–^10^. The sophisticated analytical methods for quantitative estimation of major compounds of this plant have also been reported^11^–^16^.

*Eclipta alba* (Asteraceae) is an another traditional medicinal plant known as *Bhringaraj*. The plant is an active ingredient of many herbal formulations prescribed for liver ailments and shows effect on liver cell generation^17^. It is used in treating enlargement of the liver and spleen and in various chronic skin diseases traditionally^18^. This plant has been well reported to exhibit hepatoprotective activity^19^ and in the treatment of infective hepatitis^20^ is reported in a clinical study. *In vivo* hepatoprotective activity of alcoholic extract^23^–^24^ and analgesic study of total alkaloids of *Eclipta alba* have also been reported^25^. The leaves of *Eclipta alba* showed antihyperglycemic activity^21^. The roots of *Eclipta alba* were found effective in wound healing^22^. A number of compounds had been isolated from the plant. Wedelolactone and de-methyl wedelolactone, complex furanocoumarins have been identified as its hepatoprotective principles^26^. Literature survey revealed that HPLC^26^ and UV spectrophotometry^27^ methods had been reported for the estimation of wedelolactone in a methanol extract.

*Andrographis paniculata* and *Eclipta alba* are generally found in various marketed polyherbal and traditional formulations utilized in treatment of hepatic disorders. This study details the development of an analytical method for the simultaneous estimation of the andrographolide and wedelolactone as markers of the individual drugs in herbal formulations.

All the solvents and reagents of analytical grade purchased from M/s.Qualigens India Ltd. were used. The solvents were redistilled before use. The precoated silica gel plates (TLC plates, silica gel aluminium sheets with 60 F-254) were purchased from M/s. Merck India Ltd.

The pre coated silica plates were first washed with methanol and then activated at 60° for 10 min prior to application of samples which were spotted at the application rate of 150 nl/s in the form of bands using Linomet V (Camag) applicator. The plates were allowed to develop in toluene: acetone: formic acid (9:6:1, v/v/v), as mobile phase. The optimized chamber saturation time was 35 min at room temperature (25±2°). The plates were allowed to dry at room temperature (25±2°) at relative humidity of 60±5%. The dried plates were scanned and quantified in reflectance- absorbance mode at 254 nm using the Camag TLC Scanner-3.

Standard stock solutions of andrographolide (200 μg/ml) and wedelolactone (100 μg/ml) were prepared in methanol separately. These were then diluted with methanol and mixed to obtain a standard mixture of 40 μg/ml of andrographolide and 20 μg/ml of wedelolactone. Different volumes of the standard mixture to cover a range of 200-400 ng/spot and 100-200 ng/spot of andrographolide and wedelolactone respectively, were spotted five times separately on the TLC plate.

Two marketed formulations, tablet and syrup (Vasuliv tablet and Vasuliv syrup, Vasu Pharmaceuticals Pvt. Ltd., Vadodara, Gujarat) containing both *Andrographis paniculata* and *Eclipta alba* being one of the ingredients were procured from the retail market. The test samples were prepared by sonicating 100 mg of powdered tablets in 25 ml of methanol for 30 min. The mixture was then centrifuged; 10 ml of supernatant was reduced to a volume of 2 ml which was diluted with methanol to make final volume of 5 ml to be used for further studies. Similarly, 10 ml of syrup was shaken vigorously for 10 min with methanolic solution of sodium carboxy methyl cellulose (1.4 g/ml) in separating funnel to adsorb out the additives. The mixture was then treated with n-butanol to extract both the analyte. The butanol extract was then evaporated to dryness using rotary vacuum evaporator. The dried mass was then dissolved in minimum amount of methanol and diluted with methanol to make 10 ml. The procedure was monitored continuously using TLC at the end of each step to check complete extraction of both andrographolide and wedelolactone.

The specificity of the method was ascertained by comparing the R_f_ and spectra of the spot of test and standard samples of andrographolide and wedelolactone. The validation parameters for the proposed method are shown in Table 1. The recovery of the drug at different levels in the formulations was checked by first spotting the test samples of known concentration of andrographolide and wedelolactone on the plates. The spots were then spiked in three different concentrations (80%, 100% and 120% w/w) by further adding known amount of standard mixture of andrographolide and wedelolactone. These samples were then analyzed using the same set up of instrument as in case of the estimation of the test samples.

**TABLE 1:** VALIDATION PARAMETERS FOR ANDROGRAPHOLIDE AND WEDELOLACTONE BY HPTLC

Parameters	Values	
	
	Andrographolide	Wedelolactone
Linearity range (ng/spot)	200 - 400	100 - 200
Correlation coefficient	0.9989	0.9972
Regression equation (y=mx+c)	Y = 2.405 X - 30.376	Y = 3.691 X - 299.17
Recovery studies^a^		
80% level	98.11±0.08	99.94±0.11
100% level	99.63±0.12	101.3±0.26
120% level	100.35±0.17	102.01±0.26
Recovery studies^b^		
80% level	99.12±0.41	100.30±0.38
100% level	98.16±0.46	101.80±0.36
120% level	99.05±0.49	102.50±0.66
Precision (%RSD)		
Intra-day (n=5)	0.63	0.40
Inter-day (n=5)	0.36	0.38
Limit of detection (LOD)	26.16 ng	5.06 ng
Limit of Quantification (LOQ)	79.28 ng	15.32 ng

a Recovery study for tablet. Each value is mean±standard deviation of three determinations.

b Recovery study for syrup. Each value is mean±standard deviation of three determinations.

The mobile phase containing toluene: acetone: formic acid (9:6:1, v/v/v) gave R_f_ 0.52 and R_f_ 0.58 for andrographolide and wedelolactone respectively. The linear regression data showed a good linear relationship over a concentration range of 200-400 ng/spot and 100-200 ng/spot of andrographolide and wedelolactone, respectively. The repeatability of sample application and measurement of the peak area was expressed in terms of % relative standard deviation. The % RSD was found to be less than 1.0 in all cases indicates no significant variations in the analysis of andrographolide at the concentration of 250, 300 and 350 ng/spot and in the analysis of wedelolactone at the concentration of 125, 150 and 175 ng/spot.

The peak purity of andrographolide and wedelolactone was assessed by comparing the spectra at peak start, peak apex and peak end position of the spot. Good correlation was also obtained between standard and sample overlain spectra of andrographolide and wedelolactone. The estimations were performed by varying the selected parameters within certain limits and there has been no notable alteration found on method performance and in results obtained. The developed method thus, can be considered as robust enough.

Analytical data representing recovery studies after known amount of pre analyzed samples were applied at three different concentration levels, afforded recovery for andrographolide and wedelolactone were more than 98%. Two spots at R_f_ 0.52 and 0.58 were observed in the chromatogram (Fig. 1) of the drug samples extracted from tablets and syrup. There was no interference in analysis of andrographolide and wedelolactone from the other components present in the sample. The content of andrographolide and wedelolactone found in tablet and syrup were listed in Table 2. The estimations were performed in triplicate and RSD was less than 2.0% in each case.

**Fig. 1:**
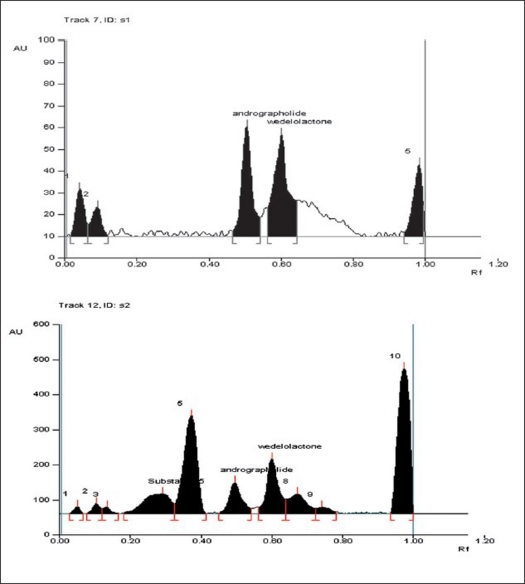
A typical HPTLC chromatogram of formulations. HPTLC chromatogram of tablet (A) and HPTLC chromatogram of syrup (B).

**TABLE 2:** QUANTITATIVE ESTIMATION IN POLYHERBAL FORMULATIONS.

Phytoconstituents	*Average Amount present (mean±SD)	
	
	Tablet (mg/tablet)	Syrup (mg/10 ml)
Andrographolide	1.45±0.14	0.56±0.05
Wedelolactone	1.66±0.15	0.39±0.03

* Each value is mean±standard deviation of three determinations.

The statistical analysis indicates that the method is repeatable, precise and selective. Thus the developed and validated HPTLC method for estimation of andrographolide and wedelolactone simultaneously in any polyherbal formulations is found accurate and robust, and therefore can be adopted for quality control of such formulation on routine basis.
